# Utilizing immunomarking techniques to track *Halyomorpha halys* (Hemiptera: Pentatomidae) movement and distribution within a peach orchard

**DOI:** 10.7717/peerj.1997

**Published:** 2016-05-11

**Authors:** Brett R. Blaauw, Vincent P. Jones, Anne L. Nielsen

**Affiliations:** 1 Department of Entomology, Rutgers, The State University of New Jersey, Bridgeton, NJ, United States; 2 Department of Entomology, Washington State University, Wenatchee, WA, United States

**Keywords:** Brown marmorated stink bug, Dispersal, Behavior, Capture, Protein marking

## Abstract

In this study we focus on the invasive brown marmorated stink bug, *Halyomorpha halys* (Stål) (Hemiptera: Pentatomidae), which has a strong dispersal capacity and has had a significant impact on several cropping systems, including peach (*Prunus persica* (L.)). Management of *H. halys* has relied on intensive insecticide use, and thus a better understanding of its dispersal behavior may assist in developing improved management strategies. In order to investigate *H. halys* movement and distribution patterns within a peach orchard we applied ecologically safe, food protein markers to the trees along the orchard border (chicken egg albumin in the form of liquid egg whites) and to the trees within the orchard interior (bovine casein in the form of cow’s milk). We used enzyme-linked immunosorbent assays (ELISA) to assess whether collected *H. halys* were “marked” with either of the two protein markers, revealing where in the orchard the bugs had visited. From the density data we determined that *H. halys* is a perimeter-driven pest in peaches, with a significantly higher density of bugs collected along the orchard border. Interestingly, this trend is primarily driven by the distribution of male bugs. The protein marking data revealed that a small proportion of male *H. halys* move equally between the orchard border and interior, while a small proportion of females move predominately to the border after visiting the interior. The verification of a strong edge-effect, although potentially sex-specific, implies that *H. halys* displays a dispersal behavior that may also be exploited for management, which may help growers more efficiently and more effectively manage *H. halys*.

## Introduction

Dispersal, as defined by an individual’s movement in space to promote gene flow (Benton & Bowler, 2012), is a behavior that is prevalent amongst organisms that impacts the spatial and temporal dynamics of populations. There are a number of reasons an organism may disperse from or to a location, including mate selection, kin competition, reduction in inbreeding, resource competition, or environmental stochasticity (Bowler & Benton, 2005; Mazzi & Dorn, 2012). Examining the natural movement and distribution of organisms in the field can be challenging, and thus studies on animal dispersal behavior or movement within a defined location frequently utilize a variety of marking techniques (i.e., mark-release-recapture, mark-capture, self-marking) (Hagler & Jackson, 2001).

Mark-release-recapture techniques artificially enhance the population by releasing marked individuals and may skew assumptions on natural dispersal behavior. Marking insects *in situ* is an approach that minimally impacts natural movement and dispersal over space and time. As a method to investigate insect dispersal and movement patterns, previous studies have relied on immunomarking (Hagler et al., 1992) where insects are “marked” with a unique protein either by direct contact during application or subsequently through contact of previously marked surfaces (Jones et al., 2006; Hagler et al., 2014). Insects that are “marked” by the protein markers can be captured and then analyzed for the specific protein by an enzyme-linked immunosorbent assay (ELISA). Jones et al. (2006) developed an ELISA procedure using low cost and easily obtainable food proteins, such as chicken egg albumin (egg whites) and bovine casein (cow’s milk). This method allows for marking naturally occurring insects in the field using highly sensitive, inexpensive, and ecologically safe markers. It has since been used to study the natural dispersal and movement patterns of a variety of insects, including natural enemies (Horton, Jones & Unruh, 2009; Swezey et al., 2014) and herbivores (Sivakoff, Rosenheim & Hagler, 2012; Reisig, Roe & Dhammi, 2013; Swezey et al., 2013).

Marking studies in agricultural settings allows the identification of source–sink dynamics, edge effects, and/or distribution patterns. By utilizing the knowledge of how an organism moves through and within an environment, we can enhance conservation through manipulating the dispersal of an organism using habitat corridors (Mönkkönen, 1999). In addition, dispersal can be limited or hindered, such as through the use of trap/barrier crops (Shelton & Badenes-Perez, 2006; Swezey et al., 2013; Swezey et al., 2014) or by employing attractants that can be used to concentrate beneficial insects in such a manner as increase biological control in a portion of the habitat (Kaplan, 2012). The identification of a strong edge-effect from an organism dispersing from one area to another indicates a perimeter-driven species, a behavior which may also be exploited for management. For example, in an agricultural landscape, perimeter trapping can be an effective tool to manage apple maggots, *Rhagoletis pomonella* (Walsh) (Diptera: Tephritidae), as adults disperse into apple orchards from the surrounding natural habitat (Bostanian et al., 1999). Similarly, border-focused applications of insecticides in tree fruit have been used to successfully manage dispersing plum curculio, *Conotrachelus nenuphar* (Herbst) (Coleoptera: Curculionidae) (Vincent et al., 1997) and brown marmorated stink bug, *Halyomorpha halys* (Stål) (Hemiptera: Pentatomidae) (Blaauw, Polk & Nielsen, 2015). Feeding injury by *H. halys* is concentrated on crop borders (Joseph et al., in press; Venugopal et al., 2014), suggesting a strong edge-effect that might be the result of immigrating individuals stopping at the crop border before dispersing inward.

*Halyomorpha halys* has more than a hundred reported host plants, including many economically important crops species, such as peach (*Prunus persica* (L.)), apple (*Malus domestica* Borkh), and soybean *Glycine max* L. Merrill (Bergmann et al., 2015). This stink bug also has a strong capacity for flight (Lee & Leskey, 2015; Wiman et al., 2015) which likely aids its dispersal ability amongst host crops. This ability may be important for the development and survival of *H. halys*, as its host preference appears to change throughout the season (Nielsen & Hamilton, 2009a). Some crops, such as soybean, are not colonized by *H. halys* until later in season, with peak population abundance occurring when pods begin to fill (Nielsen, Hamilton & Shearer, 2011). Similarly, apples often experiences high populations and heavy damage from *H. halys* late in the season, but otherwise is a poor host for development (Funayama, 2004; Nielsen & Hamilton, 2009b; Joseph et al., 2015). Conversely, the peach is a unique host in that it can support development of *H. halys* populations (nymphs through adults) from mid-late May through harvest (Nielsen & Hamilton, 2009b). The long susceptible period of the peach is thought to be responsible for the 80% crop loss experienced by peach growers throughout the mid-Atlantic production area in 2010 (Leskey & Hamilton, 2010). High population densities of the extremely mobile *H. halys* have forced growers, who traditionally did not manage stink bugs, to repeat treatments because most available insecticides are not effective after 3–7 days (Leskey et al., 2012b). The short residual control has resulted in peach growers applying insecticides (pyrethroids and neonicotinoids) on a 7–10 day cycle which undermines IPM programs developed over the last 40 years (Polk, Schmitt & Atanassov, 2010). Moreover, the frequent pesticide applications increase the cost of control up to four times compared to previous rates (Leskey et al., 2012c) and reduce natural enemies that suppress secondary pests, making the new management practices neither economically nor environmentally sustainable. Although population density has fluctuated since the high observed in 2010, it is clear that *H. halys* has established itself as a key pest of peach production.

A better understanding of an insect pest’s dispersal behavior using mark-recapture techniques may assist in developing improved management strategies. The objectives of this study were to use immunomarking to investigate whether *H. halys* is a perimeter driven pest with high densities of stink bugs observed along the border, and secondly whether it moves readily between the border and interior in peach orchards.

## Materials & Methods

### Study site

A field study was conducted in 2013 in a rectangular research block of mixed peach-nectarine orchard of 420 trees at 6.1 × 6.1 m spacing (1.56 ha) at the Rutgers Agriculture Research and Extension Center in Bridgeton, NJ. A forested edge on the North, soybeans on the East, an apple orchard on the South, and a mixed varietal peach orchard on the West bordered the research orchard.

### Insect marking procedure

In order to mark naturally occurring stink bugs, two unique protein marking solutions were applied to the orchard at four different times throughout the season: 20 May, 30 May, 12 July, and 30 July. The two unique protein marking solutions were a 5% liquid egg white solution (AllWhites^®^, Michael Foods, Inc., Minnetonka, MN) and a 20% milk solution (Provident Pantry™, Emergency Essentials, LLC, Orem, UT), both diluted in tap water (Jones et al., 2006; Jones, Melton & Baker, 2011). Additionally 0.3 g/L of sodium ethylenediamine tetra acetate (EDTA; S25311; Thermo Fisher Scientific, Waltham, MA, USA), and 1,300 ppm of Silwet L-77 (Momentive Performance Materials Inc., Columbus, OH), were added to the marking solutions to reduce water hardness and enhance the distribution and residual time of the solutions (Jones et al., 2006).

The protein solutions were applied to the orchard with a tractor-driven Pak-Blast airblast sprayer (Rears Mfg Co., Eugene, OR) at a rate of 935 L/ha. The egg white solution was applied to only the edge trees (“border”) along the orchard perimeter (76 trees), whereas the milk solution was applied to the orchard “interior” (216 trees), leaving a two tree row buffer zone between the “border” and “interior” application areas (Fig. S1).

### Insect sampling procedure

Identification of the distribution and location of *H. halys* within the orchard was conducted by sampling for stink bug adults at 16 sites (eight along the border and eight in the interior) within the orchard (Fig. S1). The eight sites were distributed with two sampling sites along each side of the orchard, which corresponded to interior sampling sites that were 48 m deep into the orchard interior. At each of these sites, three trees were sampled using beat sampling to dislodge bugs onto a flat, 20.3 by 30.5 cm sticky sheet of cardstock coated with a thin layer of Tangle-Trap (Tanglefoot, Contech Enterprises, British Columbia, Canada). Collected *H. halys* adults were immediately removed from the sticky sheet with a clean toothpick (to reduce potential protein contamination), sex was recorded and then the individual was placed in a 1.5 ml microcentrifuge tube and frozen for later analyses by the ELISAs described below. Stink bugs were sampled 1, 3, 5, and 7 days after the proteins were applied to the orchard. Additionally, stink bugs were collected from other orchards prior to protein application in order to be used as negative controls.

### Leaf sampling procedure

In order to assess the uniformity of protein application to the trees, we collected leaves from the 16 insect sampling sites sample within the orchard. After the protein solution was dry and again 4–6 days later, three leaves from each of three trees were randomly collected and combined in individual plastic bags for each sampling sites. In the laboratory, a 7-mm-diameter leaf disc was removed with a cork borer (cleaned with 70% ethanol after each use) from one of the randomly chosen leaves from each of the 16 sampling sites. Leaf discs were placed in individual 1.5 ml microcentrifuge tubes and frozen for later analyses by the ELISAs described below. Negative controls for leaves were collected following the same protocols prior to protein application to the orchard.

### Protein assessment procedure

Separate immunoassays were performed as indirect enzyme-linked immunosorbent assays (ELISA), following slightly modified methods from Jones et al. (2006), to detect for the presence of egg white or milk protein on the field-collected stink bugs and leaf discs. Commercially available antibodies for chicken egg albumin, such as rabbit anti-egg (C6534, Sigma-Aldrich, St. Louis, MO, USA), and bovine casein, rabbit anti-casein (bs-0813R; Bioss Inc., Woburn, MA, USA), were used. The secondary anti-body used for both the egg white and milk assays were peroxidase conjugated (31503; Pierce Biotechnology, Rockford, IL, USA) donkey, anti-rabbit IgG (H + L) (SAB3700926; Sigma-Aldrich).

One ml of the extraction buffer solution, tris-buffered saline (TBS, pH 8.0; T6664; Sigma-Aldrich) plus 0.3 g/liter EDTA, was added to each sample tube, and stink bugs and leaf discs, including negative control samples, were soaked for 3 min and then discarded. An 80 µl aliquot of each sample was transferred via pipette into individual wells of a 96-well microplate (Nunc-Immuno™ MaxiSorp™; Thermo Fisher Scientific). Each 96 well microplate (8 × 12 wells) was laid out as follows: first column (eight wells) was extraction buffer only, second column was negative control samples, columns three through ten were the samples to be tested, column eleven was deionized water, and column twelve was the positive control samples (5% egg white or 20% milk solution). Samples were incubated at 37 °C for 2 h on an orbital plate shaker (Standard Orbital Shaker, Model 3500, VWR International) and the contents of each well were then discarded.

For both the egg white and milk assays, the microplates were washed five times with 300 µl/well phosphate buffered saline (PBS; P4417; Sigma-Aldrich) plus 0.09% Triton-X100 (X100; Sigma-Aldrich) (PBST). Then 300 µl/well of blocker solution was added to the microplates, which was composed of PBS plus 1,300 ppm Silwet L-77 (Momentive Performance Materials Inc., Columbus, OH) plus 20% bovine serum (B-9433; Sigma-Aldrich) for the egg white assays or 10% ethanolamine (E9508; Sigma-Aldrich) for the milk assays. After blocking for 1 h, the microplates were washed twice with 300 µl/well of the PBST solution, and 80 µl/well of the diluted primary antibodies were added. The primary antibodies were diluted at a ratio of 1:6,000 for the egg white assay and 1:1,000 for the milk assay in a solution of PBS plus 1,300 ppm Silwet L-77 and 20% bovine serum. The secondary antibodies were diluted in the same solution at a ratio of 1:28,000 for the egg white assay and 1:20,000 for the milk assay (all antibody dilutions were determined using a checkerboard titration assay (Crowther, 2001)). The primary antibodies for both egg white and milk assays were incubated for 30 min and were then discarded. The microplates were washed five times with 300 µl/well PBST, then 80 µl/well of secondary antibodies were added, and then were allowed to incubate for 2 h. After incubation, the secondary antibody was discarded, and the microplates were washed three times with 300 µl/well PBS plus 2.3 g/liter sodium dodecyl sulfate (SDS; L-4509; Sigma-Aldrich) (PBS-SDS), followed by three more washes with 300 µl/well PBST. Afterward, 80 µl/well of Ultra-TMB substrate solution (34028; Thermo Fisher Scientific) was added to each well, and the microplates were incubated in the dark at room temperature for 5 min for the egg white assay and 10 min for the milk assay. After incubation, 80 µl/well of 2 N H_2_SO_4_ (258105; Sigma-Aldrich) was added to stop the reaction. The optical density (OD) for each sample was measured with a BioTek Synergy™ 4 microplate reader (BioTek Instruments, Inc., Winooski, VT, USA) at 450 nm, using 490 nm as the reference standard. All samples were scored positive for the presence of the protein marker if the ELISA OD reading was three standard deviations greater than the mean negative control result (Hagler & Jones, 2010).

### Analysis

The number of marked leaves for each protein (egg white and milk) were pooled separately for each location (border and interior) and replicated by sample period. The percentages of leaf samples per location that were marked with only egg white, only milk, or both proteins were compared separately against zero using a one sample *t*-test.

Adult densities were compared between orchard border and interior locations using a generalized linear mixed model (GLMM). The densities of bugs were pooled for each of the 16 sample sites for each of the sampling periods (20 May, 30 May, 12 July, and 30 July). The single fixed factor was sample location blocked by sampling period as the random factor, with a Poisson distribution and log link function. This was repeated to compare density of bugs collected between each location for each sex. The total number of females and males collected were pooled separately for the entire season for each of the 16 sampling sites and the means were compared using the nonparametric Kruskal–Wallis test blocking by sampling period with chi-square approximation, after data did not meet assumptions of normality. Additionally, to assess the distribution and movement of all collected *H. halys* during the seven days after protein application, the total densities of collected *H. halys* were pooled separately for each orchard location, averaged across sampling periods, and compared between the two locations for each of the four sampling days (1, 3, 5, and 7) using the nonparametric Kruskal–Wallis test with chi-square approximation. Furthermore, in order to review the longevity of the egg and milk proteins, we plotted the mean percentages of all bugs marked with egg only, milk only, or both proteins, averaged across sampling periods for each of the four sampling days.

The densities of positively marked stink bugs collected per sampling site were compared between crop border and interior locations using a GLMM. Bugs marked with either egg white only, milk only, or both proteins were pooled separately for each sampling site and sampling period. The single fixed factor was sample location (border or interior) blocked by sampling period as a random factor, with a Poisson distribution and log link function. The densities of positively marked stink bugs were summed for each location/sample period to calculate the percentage of marked bugs along the border and within the interior of the orchard. We compared the percentages (arcsine transformed) of positively marked specimens between crop border and interior locations using a one-way analysis of variance (ANOVA), blocked by sampling period as the random factor. This was repeated to compare percentage of marked bugs collected between each location, separately for each sex.

As all the collected bugs did not test positive for proteins through the ELISA analysis, additional statistical analyses were performed on “corrected” percentages. To better represent movement between border and interior, *H. halys* adults were assumed to be positively marked with the appropriate protein if collected within the respective orchard location regardless of ELISA results. For example, if a specimen was collected along the crop border, but tested only positive for milk protein, that stink bug was corrected to be marked as “both” because the border was treated with egg white protein. The corrected percentages of positively marked stink bugs between crop border and interior locations were then compared using an ANOVA as described above. This was repeated separately for males and females. Statistical analyses were performed with SPSS v.20.0 (IBM Corp., Armonk, NY).

## Results

The ELISA analysis of the 64 leaf samples collected from each orchard location (border and interior) revealed that the majority of the leaves along the orchard border and within the orchard interior were successfully marked with their respective protein marker (Table 1). Marking efficacy of the orchard varied between the two protein markers, and was generally lower with the milk protein, with 31–81% marked versus 62–94% marked with egg whites (Table 1), which may have been due to application coverage or the efficacy of the ELISA detection assays (Hagler et al., 2014). Some sample contamination did occur either from drift during application or other potential, unknown cross-contamination of samples, but the percentage of contaminated samples was not significantly greater than zero (Table 1).

**Table 1:** Mean optical density (±SD) for all samples that tested positive for the protein marker and mean (±SEM) percentage of leaves marked positive with egg white, milk, or both protein marker solutions (averaged across four sampling periods) collected from the orchard border and interior, and compared to zero with a one sample *t*-test. 64 leaves were collected per location.

Protein^a^	Orchard Location	Optical density	*n* positive	% marked positive	*t*	DF	*P*
Egg white	Border	0.342 ± 0.052	49	79.7 ± 6.0	11.5	3	0.001
	Interior	0.313 ± 0.046	5	12.5 ± 4.9	2.2	3	0.116
	− control	0.036 ± 0.002	40^b^				
	+ control	0.328 ± 0.036	40				
Milk	Border	0.138	1	1.6 ± 0.9	1	3	0.391
	Interior	0.102 ± 0.055	32	53.1 ± 10.7	4.9	3	0.016
	− control	0.037 ± 0.003	40^b^				
	+ control	0.229 ± 0.052	40				
Both	Border	Egg: 0.2364 ± 0.015	2	1.6 ± 0.9	1	3	0.391
		Milk: 0.179 ± 0.042					
	Interior	Egg: 0.291 ± 0.017	3	3.1 ± 2.0	1	3	0.391
		Milk: 0.179 ± 0.042					

**Notes.**

a Border was marked with only egg white and the interior with only milk.

b 40 samples tested, none were positive.

**Figure 1:**
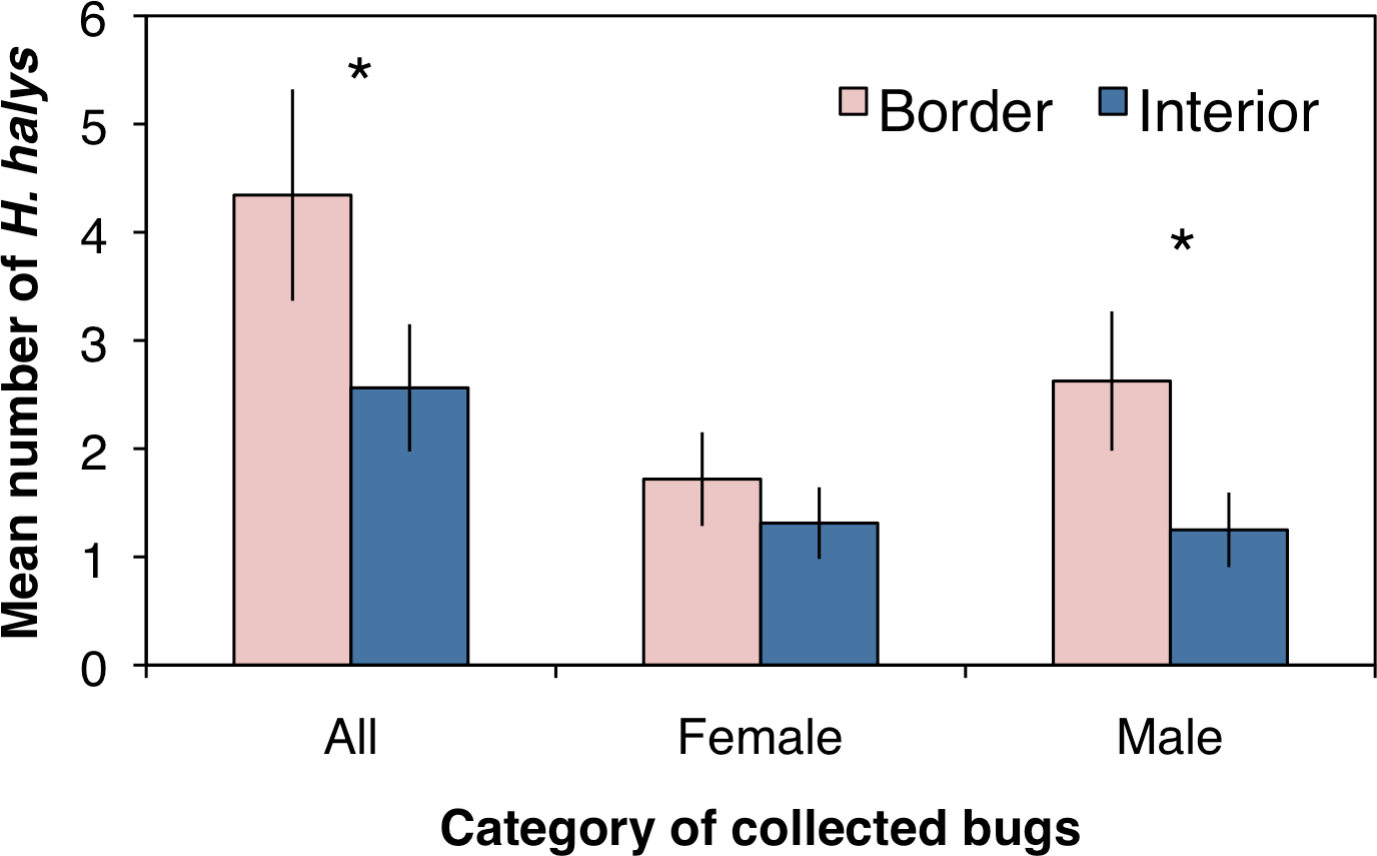
Mean (±SEM) density of *H. halys* collected in peach orchards comparing separately all, only female, and only male bugs between the orchard border and interior. Asterisks indicate significant difference (GLMM; *P* < 0.05).

A total of 221 adult *H. halys* were collected across all sampling sites in the peach orchard, with 140 collected along the crop border and 81 from the interior. Over the entire season there were significantly more stink bugs collected along the orchard border than the orchard interior (Fig. 1; *F*_1,62_ = 14.4, *P* < 0.001). Of the *H. halys* adults collected, there was no difference in the average abundance (±SEM) of females (1.51 ± 0.27) compared to males (1.94 ± 0.37) (*χ*^2^ = 0.21, df = 1, *P* = 0.644). However, the distribution of *H. halys* within a peach orchard may be influenced by the location of male bugs. Female *H. halys* were evenly distributed between the border and interior, with no significant difference in the density of female bugs collected between locations (Fig. 1; *F*_1,62_ = 1.73, *P* = 0.193), whereas significantly more males were collected along the orchard border compared to the interior (Fig. 1; *F*_1,62_ = 14.92, *P* < 0.001).

**Figure 2:**
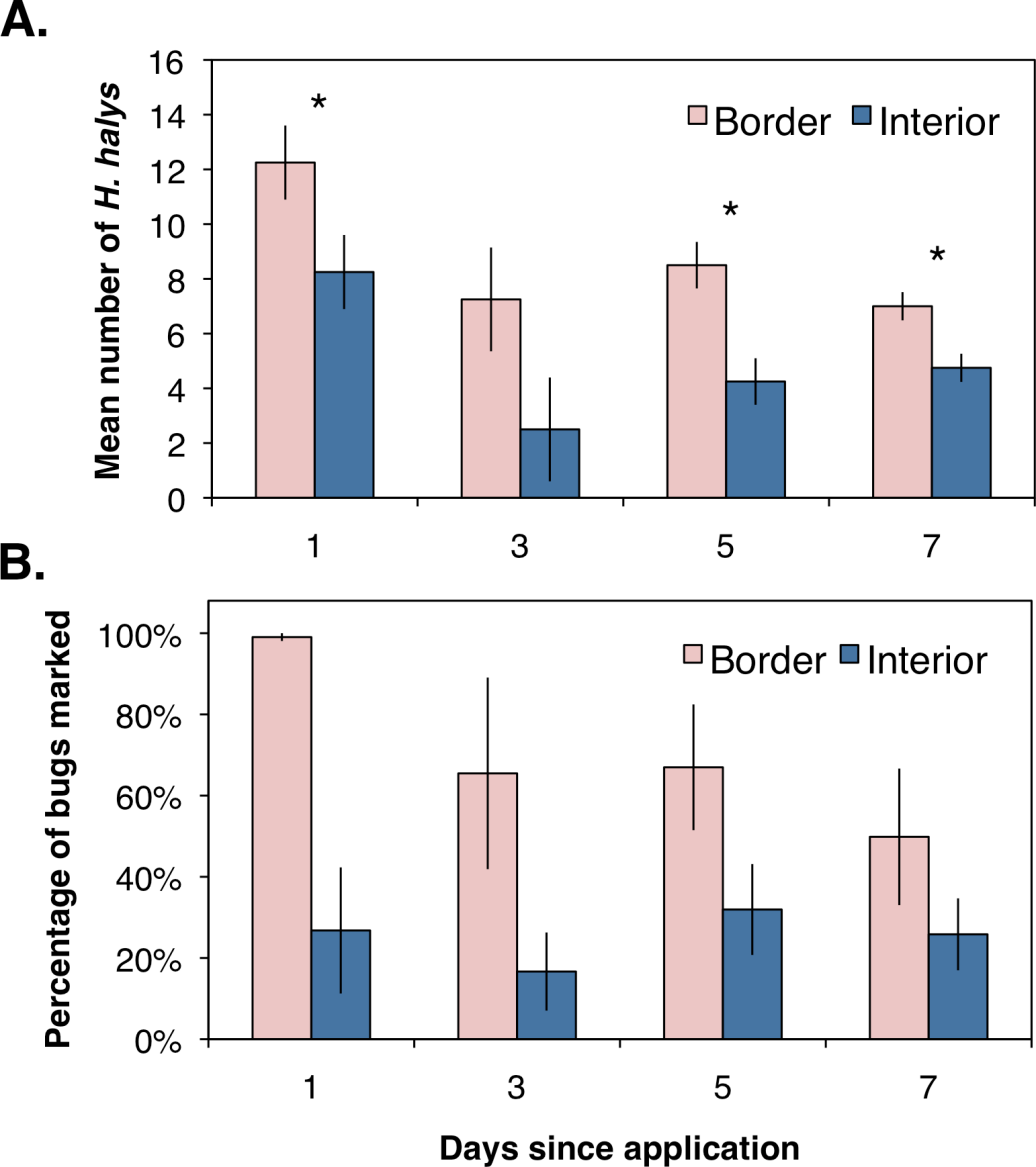
(A) Mean (±SEM) density of all *H. halys* collected in peach orchards comparing those collected from the orchard border and interior over seven days of sampling. Asterisks indicate significant difference (Kruskal–Wallis; *P* < 0.05). (B) Mean (± SEM) percentage of bugs marked positive for both proteins, indicating the longevity of the protein markers in the field.

Assessing the distribution and movement of all collected *H. halys* revealed that after the first day of sampling there were significantly more bugs collected along the orchard border than within the interior (Fig. 2A; *χ*^2^ = 4.86, df = 1, *P* = 0.027). Even though an average of 12 bugs were removed from each location during the first day of sampling, we were able to continue collecting bugs the following days, suggesting that *H. halys* continually colonizes peach orchards. The abundance of bugs collected by the third day dropped for both locations with no significant difference between the two (Fig. 2A; *χ*^2^ = 2.08, df = 1, *P* = 0.149). By the fifth and seventh days, there were significantly higher numbers of bugs collected along the crop border (Fig. 2A; *χ*^2^ = 5.33, df = 1, *P* = 0.021; *χ*^2^ = 4.86, df = 1, *P* = 0.027, respectively). Additionally, even though percentages of positively marked *H. halys* fluctuated over the seven day period, the egg and milk proteins were persistent enough in the orchard that we were able to consistently collect bugs marked with either egg only, milk only, or both proteins across the seven day period (Fig. 2B).

From the 221 adult *H. halys* that were collected, 87 were marked with egg white only (border), 32 with milk only (interior), and 21 with both protein markers. There was a significant difference in the mean density (±SEM) of stink bugs marked with egg white only per sampling site along the border (2.5 ± 0.7) compared with those collected within the interior (0.22 ± 0.07; *F*_1,62_ = 38.1, *P* < 0.001). Although overall fewer bugs were marked with only milk, significantly more were marked with milk per sampling site that were collected from the interior (0.72 ± 0.22) than from along the edge (0.28 ± 0.11; *F*_1,62_ = 4.65, *P* = 0.035). There were more stink bugs marked with both proteins collected per sampling site along the border (0.5 ± 0.16) compared with those collected within the interior (0.16 ± 0.08), but this difference was not statistically significant (*F*_1,62_ = 2.36, *P* = 0.131).

When analyzing the uncorrected percentages of marked *H. halys*, we found that a total of 63.3% were successfully marked. As with the density of all stink bugs collected, a significantly higher percentage of *H. halys* collected along the orchard border were marked with only egg white proteins compared to those collected within the interior (*P* = 0.024; Table 2). Of the bugs marked with milk, there were more collected in the interior than on the border but the results were not statistically significant (*P* = 0.061; Table 2). There was also no significant difference in the percentages of all bugs marked with both proteins for either orchard location (*P* = 0.133; Table 2). Analysis of percent marked bugs by sex revealed that for both female and male *H. halys,* the trend is consistent, and there were significantly higher percentages of bugs marked with egg whites only collected along the orchard border (Female *P* = 0.041; Male *P* = 0.013; Table 2).

**Table 2:** Mean optical density (±SD) for all samples that tested positive for the protein marker and mean (±SEM) percentage of *H. halys* adults per sampling site marked positive with egg white, milk, or both protein marker solutions compared from between orchard border and interior with an ANOVA for all, female only, and male only. Total number of bugs collected was 221.

Collected bugs	Protein^a^	Orchard location	*n* positive	Optical density	% marked positive	*F*	DF1	DF2	*P*
All	Egg white	Border	80	0.238 ± 0.142	59.3 ± 10.1	18.05	1	6	0.024
		Interior	7	0.134 ± 0.032	6.6 ± 2.5				
		− control	32^b^	0.068 ± 0.013					
		+ control	32	0.485 ± 0.139					
	Milk	Border	9	0.101 ± 0.039	7.2 ± 5.4	8.66	1	6	0.061
		Interior	23	0.101 ± 0.039	28.5 ± 2.9				
		− control	32^b^	0.043 ± 0.002					
		+ control	32	0.176 ± 0.054					
	Both	Border	16	Egg: 0.225 ± 0.116	10.3 ± 1.8	4.21	1	6	0.133
				Milk: 0.102 ± 0.045					
		Interior	5	Egg: 0.104 ± 0.022	4.5 ± 3.6				
				Milk: 0.086 ± 0.030					
Female	Egg white	Border	29	0.218 ± 0.126	58.1 ± 11.5	12.04	1	6	0.041
		Interior	3	0.107 ± 0.021	5.9 ± 2.2				
	Milk	Border	3	0.074 ± 0.018	9.5 ± 8.1	1.14	1	6	0.364
		Interior	13	0.107 ± 0.042	23.8 ± 9.6				
	Both	Border	11	Egg: 0.246 ± 0.135	15.5 ± 5.8	8.39	1	6	0.063
				Milk: 0.106 ± 0.040					
		Interior	2	Egg: 0.091 ± 0.010	3.6 ± 2.2				
				Milk: 0.096 ± 0.035					
Male	Egg white	Border	51	0.252 ± 0.149	61.1 ± 10.8	28.44	1	6	0.013
		Interior	4	0.160 ± 0.008	6.3 ± 3.7				
	Milk	Border	6	0.114 ± 0.040	6.8 ± 4.6	4.75	1	6	0.117
		Interior	10	0.094 ± 0.036	35.9 ± 11.7				
	Both	Border	5	Egg: 0.181 ± 0.033	5.1 ± 2.2	0.003	1	6	0 .996
				Milk: 0.104 ± 0.61					
		Interior	3	Egg: 0.113 ± 0.026	5.0 ± 5.1				
				Milk: 0.079 ± 0.031					

**Notes.**

a Border was marked with only egg white and the interior with only milk.

b 32 samples tested, none were positive.

**Figure 3:**
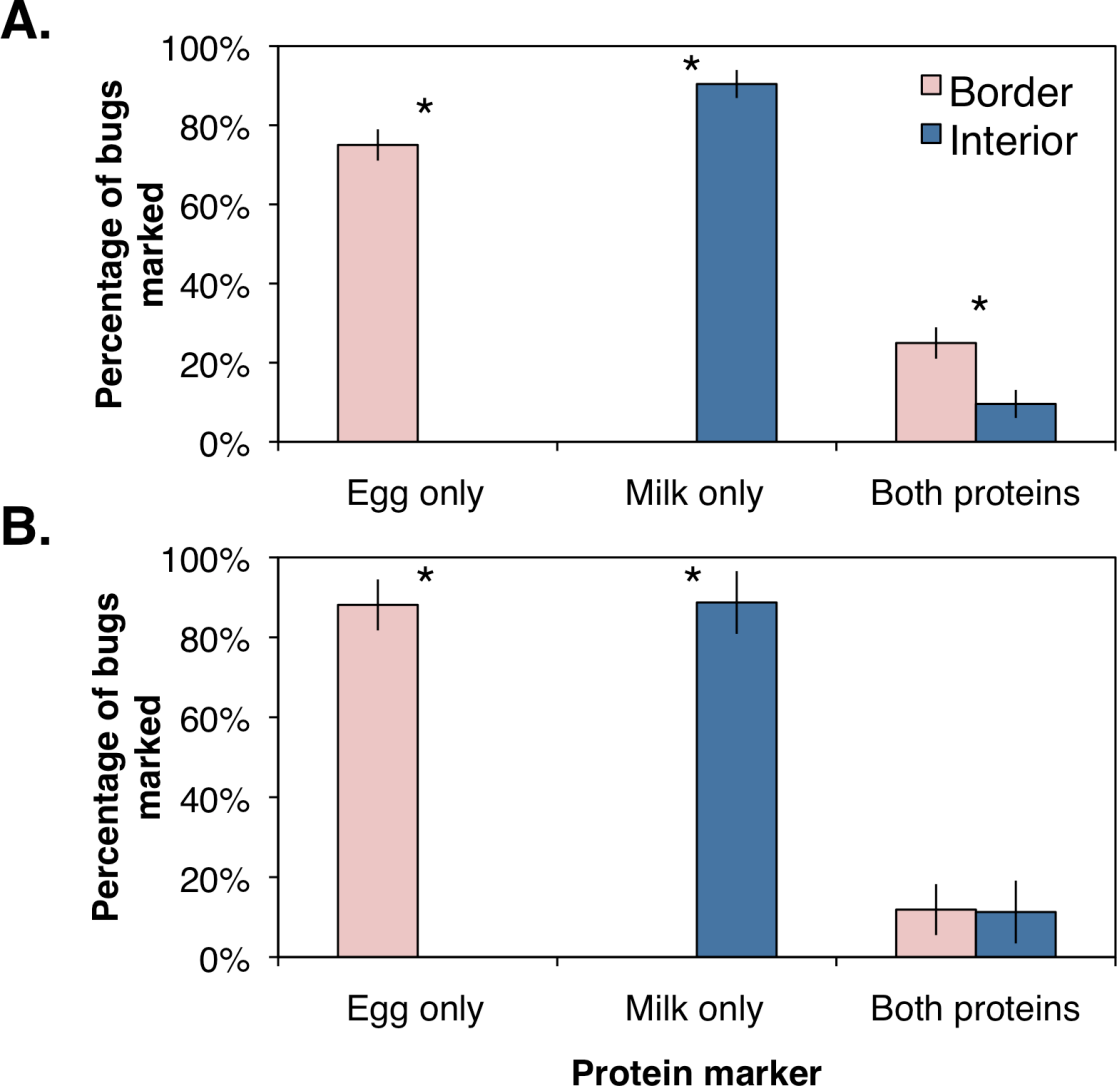
Mean (±SEM) percentage of *H. halys* adults per sampling site marked positive with egg white, milk, or both protein marker solutions compared from between orchard border and interior with an ANOVA for (A) female and (B) male bugs. Asterisks indicate significant difference (Kruskal–Wallis; *P* < 0.05).

The percentages of marked bugs were corrected for marking efficiency by assuming bugs were positively marked with a protein if collected within the respective orchard location. Using the corrected values, female and male *H. halys* were significantly more abundant along the orchard border (Female: *F*_1,6_ = 183.9, *P* = 0.001; Male: *F*_1,6_ = 51.1, *P* = 0.006; Fig. 3) than the interior. Similarly, females and males marked with milk were significantly higher in the orchard interior (Female: *F*_1,6_ = 82.9, *P* = 0.003; Male: *F*_1,6_ = 36.5, *P* = 0.009; Fig. 3). Females marked with both proteins, indicating dispersal within the orchard, was significantly higher along the border (Fig. 3; *F*_1,6_ = 25.2, *P* = 0.015; Fig. 3A), whereas males marked with both proteins were collected equally between the border and the interior (Fig. 3; *F*_1,6_ = 0.002, *P* = 0.964; Fig. 3B). These data imply that a small proportion of male *H. halys* move evenly back-and-forth between the orchard border and interior, while a small yet significant proportion of females move to the border once they have reached the interior.

## Discussion

Studies on insect dispersal, especially those utilizing marked individuals, have focused on movement between resources (i.e., crop or prey). We studied within crop dispersal to investigate how an insect species may utilize different aspects of a monoculture. An understanding of this behavior has implications for density-dependent interactions, but can also be exploited for management. Through this approach we were able to determine that *H. halys* individuals exhibit a strong edge effect in peaches. Specifically, as *H. halys* adults disperse to a highly attractive, resource filled peach orchard, they tend to arrest flight behavior along the crop border. This is similar to the dispersal patterns of *H. halys* documented in field crops (Venugopal et al., 2014), ornamentals (Venugopal et al., 2015), and vineyards (Basnet et al., 2015), and is analogous to the behavior of several native US stink bug species (Tillman et al., 2009; Tillman, 2011). The perimeter-driven nature of *H. halys* abundance in peach orchards also supports the observed patterns of higher *H. halys* crop injury along orchard borders (Leskey et al., 2012c; Joseph et al., in press).

Our data suggests that this trend is primarily driven by the distribution of male bugs, which are found in significantly higher densities along the orchard border relative to the interior whereas female densities showed no significant differences between locations. Thus, intraspecific interactions between sexes may be driving within crop distribution and dispersal. Sex-biased dispersal is not unique (Petit et al., 2001; Gros, Hovestadt & Poethke, 2008), but it remains unclear why female *H. halys* would be more likely to disperse into the interior of an resource-filled orchard. One explanation may be the preference-performance hypothesis, where females will maximize their fitness by ovipositing eggs on hosts in which their offspring will perform the best (Gripenberg et al., 2010). In a resource-filled peach orchard, this may mean that females are more likely to disperse and distribute themselves more evenly to reduce the chance of competition between offspring. This is similar to the dispersal behavior of the fritillary butterfly, *Polygonum bistorta*, where females move amongst habitat patches more often than the males, which was primarily correlated with the female population size (Petit et al., 2001). Additionally, another explanation may come from differences in behavior due to reproductive maturation in the females. For example, gravid female southern green stink bugs, *Nezara viridula* L., will disperse to different host plants, oviposit their eggs, and then re-disperse back to the original to food resource soon after oviposition (Kiritani et al., 1965) or when the secondary hosts no longer have resources because of plant phenology (Jones et al., 2001). Furthermore, the strong edge-effect observed, especially among males, could be influenced by the male-produced aggregation pheromone which is equally attractive to males and females (Khrimian et al., 2014), but the attraction to the pheromone may change throughout the photoperiod as it does with other stink bugs (Krupke, Jones & Brunner, 2006). It is unknown what impact the pheromone may have at influencing male and female retention on or dispersal between host plants.While we do not have data on the reproductive status of the collected female *H. halys* and physical sampling of peach trees only allowed us to determine the distribution of *H. halys* within the peach orchard at that specific time, the use of immunomarking, which has been used to study the dispersal and movement patterns of a variety of insects (Horton, Jones & Unruh, 2009; Sivakoff, Rosenheim & Hagler, 2012; Reisig, Roe & Dhammi, 2013; Swezey et al., 2014), allowed us to track where the stink bugs had previously been within the orchard. Unfortunately the protein marking does not clarify the process behind the differences in the distribution trend of female and male *H. halys*.

Because of the gap between sampling periods, the protein application efficiency (Table 1), and the potential for removing protein from marked bugs due to the adhesive in the collection process (Jones, Melton & Baker, 2011), we are unable to determine whether stink bugs marked with only milk protein collected from within the orchard interior were already within the orchard and were marked directly during protein application, or if they immigrated directly into the interior bypassing the orchard border.

Although *H. halys* appears to be a perimeter-driven pest, our data reveal that it readily disperses and re-invades peach orchards along the border as well as within the interior. We sampled the same three trees per sampling site over the course of a seven-day period, and even though bugs were continually removed from each sampling site location, an average of 0.5–1 bugs per site were collected each of the following seven days. Additionally, immunomarking revealed that a total of 16.7% of *H. halys* adults collected were marked (corrected for marking efficiency) with both proteins, implying that there is significant movement within the orchard. Identifying the bugs marked with both proteins revealed that female *H. halys* appear to move more readily from the orchard interior to the orchard border with over double the percentage (corrected) of females marked with both proteins collected along the border. Conversely, the density of male stink bugs were nearly double along the crop border, but the percentages (corrected) of males marked with both were nearly identical in both locations. This supports previous demonstrations that *H. halys* has a strong dispersal capacity (Lee & Leskey, 2015; Wiman et al., 2015).

The strong capacity for dispersal and its polyphagous behavior has aided *H. halys* in emerging as a key pest of many annual and perennial crops in the mid-Atlantic region of the United States. Currently, many peach growers rely on either repeated 10–14-day whole block or 7-day alternate-row-middle application of insecticides to manage *H. halys*, which has disrupted previous pest management programs (Polk, Schmitt & Atanassov, 2010; Leskey et al., 2012c). The need for frequent, repeated insecticide applications is due to the suspected continuous immigration of adults (as evidenced here) and the short residual period of effective insecticides (Nielsen, Shearer & Hamilton, 2008; Leskey et al., 2012b). Similar to *H. halys* management in soybean (Leskey et al., 2012a) and to other border-driven orchard pests, such as *C. nenuphar* (Chouinard et al., 1992; Vincent et al., 1997), exploiting this behavior through spatially-precise perimeter focused applications of insecticides is a tactic that can improve *H. halys* management while reducing insecticide inputs (Blaauw, Polk & Nielsen, 2015).

## Conclusions

Understanding the movement and distribution patterns of *H. halys* adults in an agricultural landscape will help growers more efficiently and more effectively manage *H. halys* through techniques that exploit insect behavior, such as border spray applications that will reduce overall insecticide use and help preserve beneficial insects in the agro-ecosystem. These results will be used to support further research on the effectiveness of the systems-level approach exploiting the perimeter-driven behavior of *H. halys* and to better understand the mechanisms behind the border spray approach and how it could be further optimized. Further testing, utilizing a more appropriate sampling method, a higher concentration of milk protein solution, and investigating female reproductive status is needed.

## Supplemental Information

10.7717/peerj.1997/supp-1 Data S1 BMSB movement and distribution data The raw data for the protein marking of adult *Halyomorpha halys* and peach leaves collected to assess the movement and distribution of the bugs within the peach orchard.

10.7717/peerj.1997/supp-2 Figure S1 Image of field layout and sampling map Orchard layout and sampling map, illustrating locations of protein marker application and bug collection sites. Satellite image source: 39°30′57.64″N and 75°12′1.77″W; Google Earth, 21/06/2015. Accessed 22/03/2016.

## Additional Information and Declarations

### Data Availability

The following information was supplied regarding data availability: The data is uploaded as Supplemental Information.
