# Supplementary figures and images for: Utilizing immunomarking techniques to track Halyomorpha halys (Hemiptera: Pentatomidae) movement and distribution within a peach orchard
Source: PeerJ. 2016 May 11;4:e1997. doi: 10.7717/peerj.1997 (PMC4867716; doi:10.7717/peerj.1997)

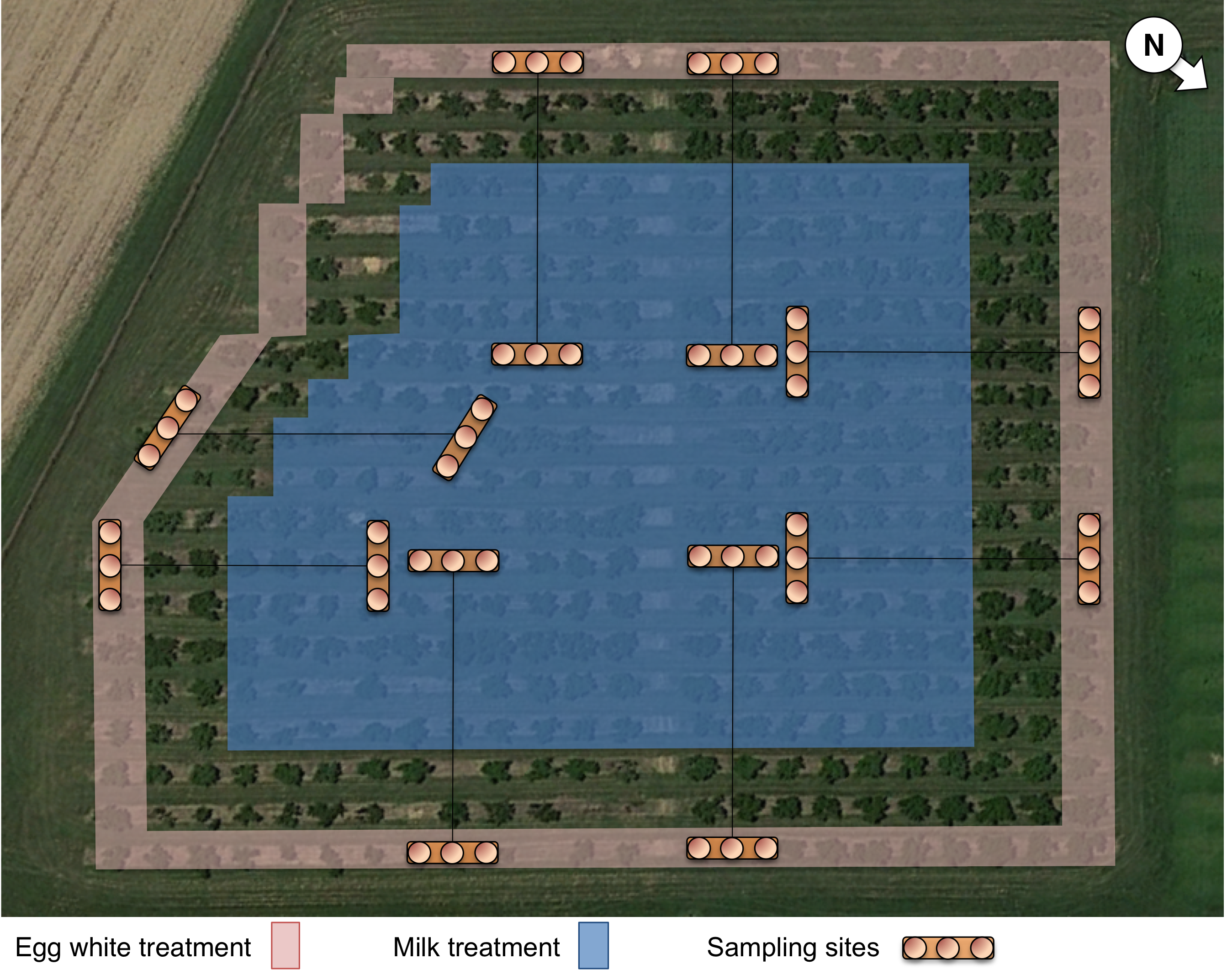

Supplement: Figure S1 — Orchard layout and sampling map, illustrating locations of protein marker application and bug collection sites. Satellite image source: 39°30′57.64″N and 75°12′1.77″W; Google Earth, 21/06/2015. Accessed 22/03/2016. [file peerj-04-1997-s002.png]
